# Design and analysis of a tunable synchronized oscillator

**DOI:** 10.1186/1754-1611-7-26

**Published:** 2013-11-18

**Authors:** Brendan M Ryback, Dorett I Odoni, Ruben GA van Heck, Youri van Nuland, Matthijn C Hesselman, Vítor AP Martins dos Santos, Mark WJ van Passel, Floor Hugenholtz

**Affiliations:** 1 Systems and Synthetic Biology, Wageningen University, Wageningen, Netherlands; 2 Laboratory of Microbiology, Wageningen University, Wageningen, Netherlands; 3 Netherlands Consortium for Systems Biology, University of Amsterdam, Amsterdam, Netherlands; 4 Centre for Zoonoses and Environmental Microbiology, Centre for Infectious Disease Control, National Institute for Public Health and the Environment (RIVM), Bilthoven, Netherlands

**Keywords:** Synchronized tunable oscillator, Genetic circuit, Transcriptional feedback, Delay differential equation

## Abstract

**Background:**

The use of *in silico* simulations as a basis for designing artificial biological systems (and experiments to characterize them) is one of the tangible differences between Synthetic Biology and “classical” Genetic Engineering. To this end, synthetic biologists have adopted approaches originating from the traditionally non-biological fields of Nonlinear Dynamics and Systems & Control Theory. However, due to the complex molecular interactions affecting the emergent properties of biological systems, mechanistic descriptions of even the simplest genetic circuits (transcriptional feedback oscillators, bi-stable switches) produced by these methods tend to be either oversimplified, or numerically intractable. More comprehensive and realistic models can be approximated by constructing “toy” genetic circuits that provide the experimenter with some degree of control over the transcriptional dynamics, and allow for experimental set-ups that generate reliable data reflecting the intracellular biochemical state in real time. To this end, we designed two genetic circuits (basic and tunable) capable of exhibiting synchronized oscillatory green fluorescent protein (GFP) expression in small populations of *Escherichia coli* cells. The functionality of the basic circuit was verified microscopically. High-level visualizations of computational simulations were analyzed to determine whether the reliability and utility of a synchronized transcriptional oscillator could be enhanced by the introduction of chemically inducible repressors.

**Results:**

Synchronized oscillations in GFP expression were repeatedly observed in chemically linked sub-populations of cells. Computational simulations predicted that the introduction of independently inducible repressors substantially broaden the range of conditions under which oscillations could occur, in addition to allowing the frequency of the oscillation to be tuned.

**Conclusions:**

The genetic circuits described here may prove to be valuable research tools for the study of synchronized transcriptional feedback loops under a variety of conditions and experimental set-ups. We further demonstrate the benefit of using abstract visualizations to discover subtle non-linear trends in complex dynamic models with large parameter spaces.

## Background

### Synthetic genetic circuits as research tools

In order for synthetic genetic circuits to be technologically useful and modularly composable in higher order systems, their properties must be subject to formal mathematical descriptions that capture the salient features of a given circuit [1]. One of the aims of synthetic biology is to develop models that are sufficiently accurate and comprehensive to provide a basis for predicting the emergent properties of newly built genetic circuits under varying conditions [2,3]. Modeling approaches based on either *a priori* mechanistic descriptions (e.g. delay differential equations) [4], and to a lesser extent, data driven “black-box” model structure identification methods (e.g. NARMAX [5]) have become increasingly prominent. More recently, approaches attempting to consolidate models operating at varying levels of biological abstraction have also been proposed [6].

One way to improve the accuracy of models is to construct “toy” circuits which have externally controllable parameters. Such systems facilitate the rapid generation of a wide range of experimental conditions, which can be modeled in order to gain insights into potentially interesting dynamic behaviors. These systems should be complex enough to provide useful insights into the nonlinear dynamics of multi-component systems, without being so complex as to create indeterminable and/or intractable parameter spaces. Simple bi-stable memory switches have been studied extensively [7] as toy circuits for model building and parameter estimation methods, as they generally fulfill the aforementioned criteria and are amenable to study with conventional fluorescent plate-reader and FACS-based experimental set-ups. While bi-stable switches can provide insights into some non-linear regulatory interactions, they are - by their very design specifications - stable.

In contrast, genetic oscillators exhibit unstable time-variant expression dynamics which can potentially provide insights into more complex (and subtle) emergent properties [8]. Thus, genetic oscillators are excellent objects for the study of biological nonlinear dynamics, as is evidenced by the abundance of published theoretical work [9-12]. A drawback of genetic oscillators is that their experimental implementation poses non-trivial practical difficulties. Measuring the gene expression of individual bacterial cells in real time over time-spans relevant to transcriptional oscillators is technically challenging [13]. Synchronization of populations, e.g. via quorum sensing, allows populations to be studied instead of individual cells, but imposes new constraints. The fact that systems governed by more unknown parameters than measurable (or controllable) variables are inherently underdetermined confounds the improvement of mechanistic models. Therefore, it is likely that the elucidation of the oscillatory dynamics emerging from (transcriptional) regulatory feedback loops could be facilitated by the introduction of simple control elements; assuming they remain orthogonal to the system’s basic circuitry and do not increase the complexity of the nonlinear interactions (or significantly affect the host cell’s metabolism). If implemented successfully, such control elements could substantially expand the range of experimental conditions used to characterize the circuit’s oscillatory dynamics.

### Combining synchronization and tunability

**Figure 1:**
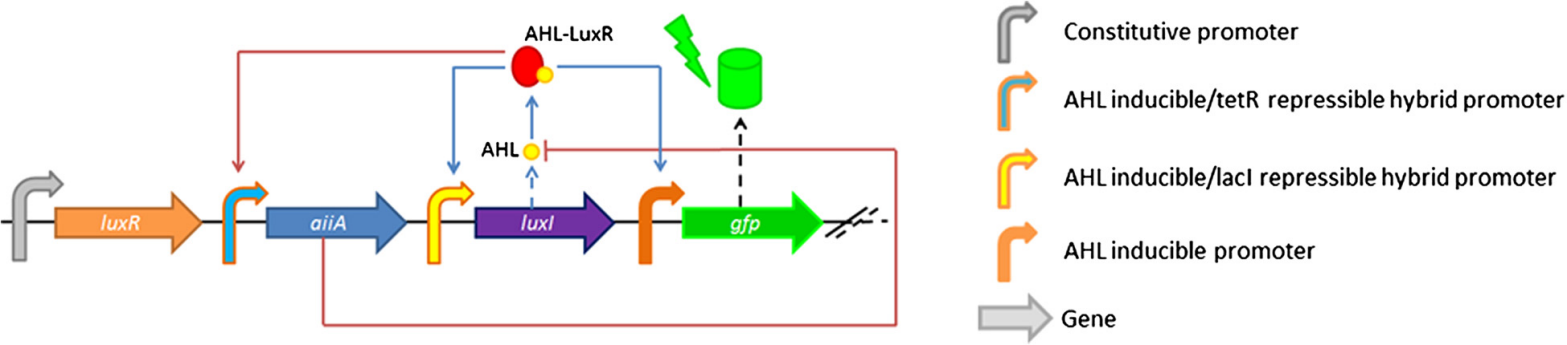
**Scheme of the basic synchronized oscillator consisting of modified lux quorum sensing machinery, aiiA, and GFP.** LuxI produces AHL, which forms a complex with the constitutively expressed LuxR. This complex induces further expression of LuxI (positive feedback loop depicted in blue) as well as AiiA, which in turn degrades AHL (negative feedback loop depicted in red). The expression of GFP is dependent on the AHL concentration present in the system and thus serves as a reporter of the oscillating AHL levels. The three AHL inducible promoters are derived from the lux promoter. Those depicted in blue and yellow are also repressible by corresponding transcription factors, but behave identically in absence of them.

To this end, we have redesigned a genetic circuit capable of producing synchronized oscillatory GFP expression in *Escherichia coli* cells. The topology and components of the basic synchronized oscillator circuit (Figure 1) were derived from a design first published by Danino et al. [14]. The circuit consists of genes encoding products that either synthesize, degrade, or respond to the presence of the quorum sensing molecule N-(3-oxohexanoyl)-homoserine lactone (hereafter referred to as AHL) and associated regulatory elements. Oscillations emerge from the coupling of positive and negative feedback loops, of which the positive feedback is based on the AHL synthase LuxI found in *Vibrio fischeri* and the negative feedback is mediated by the AHL degrading enzyme AiiA from *Bacillus thuringiensis*[14].

Transcription of the *luxR* gene is regulated by a constitutive promoter, resulting in constant levels of the AHL-dependent transcription factor LuxR. Transcription of *luxI* and *aiiA* occurs at a basal rate when intracellular AHL concentrations are below the activation threshold of LuxR. As the cell density increases in the course of normal cell divisions in a constrained space, so too does the intracellular AHL concentration. AHL diffuses freely between the cells, which effectively synchronizes their internal states. When the AHL concentration reaches the activation threshold of LuxR, the rate of transcription of *luxI* and *aiiA* is greatly increased, initially giving rise to higher levels of the enzyme LuxI due to differences in transcription and maturation time as well as ribosomal saturation [15].

This positive feedback loop results in an exponential increase in AHL synthesis, and in turn, maximal expression of LuxI and AiiA. As catalytically active AiiA accumulates, AHL is rapidly degraded (negative feedback) to sub-LuxR activation levels, and transcription of *aiiA* and *luxI* recedes to the basal rate. Oscillations arise from the delayed interaction between these coupled positive and negative feedback loops and are contingent on the ability of the system to rapidly reset to its initial state. This property is dependent on the inclusion of LVA-degradation tags in all non-constitutively expressed proteins [16]. Changes in the intracellular AHL concentration are visualized semi-quantitatively via the expression of a fluorescent reporter gene under control of a LuxR-AHL dependent promoter. Synchronization across a population of cells results from the rapid diffusion of AHL. This onset of synchronization via quorum sensing is not gradual, but sudden and a function of varying cell densities [17].

The designs used in this study differ from previously published work [14] due to three substantial modifications: (i) elimination of redundant regulatory and coding sequences, (ii) introduction of tunable hybrid promoters and (iii) consolidation of the circuit into a single DNA sequence conforming to the BioBrick assembly standard (i.e. BioBrick device) [18].

The introduction of these tuners is intended to provide an additional set of control variables that allow the kinetics of the circuit’s feedback loops to be influenced independently of one another by varying the inducer molecule concentrations within a dynamic range. This additional control may be exploited to compensate for external conditions that would prevent the basic, non-tunable circuit from producing oscillations, effectively increasing the oscillator’s robustness towards variations in cell density, and by extension, expand the range of experimental set-ups under which the circuit could be employed. The functionality of the basic, non-tunable circuit in *E. coli* was verified experimentally using a custom microbial growth chamber [19] in conjunction with a fluorescence microscope.

The dynamics of both circuits are described by a set of delay differential equations which served as the basis for deterministic simulations. A broad range of input values was chosen in order to elucidate the extent to which changes in the inducer molecule concentrations influence the cell-density dependent expression dynamics.

## Results and discussion

### Circuit design

**Figure 2:**
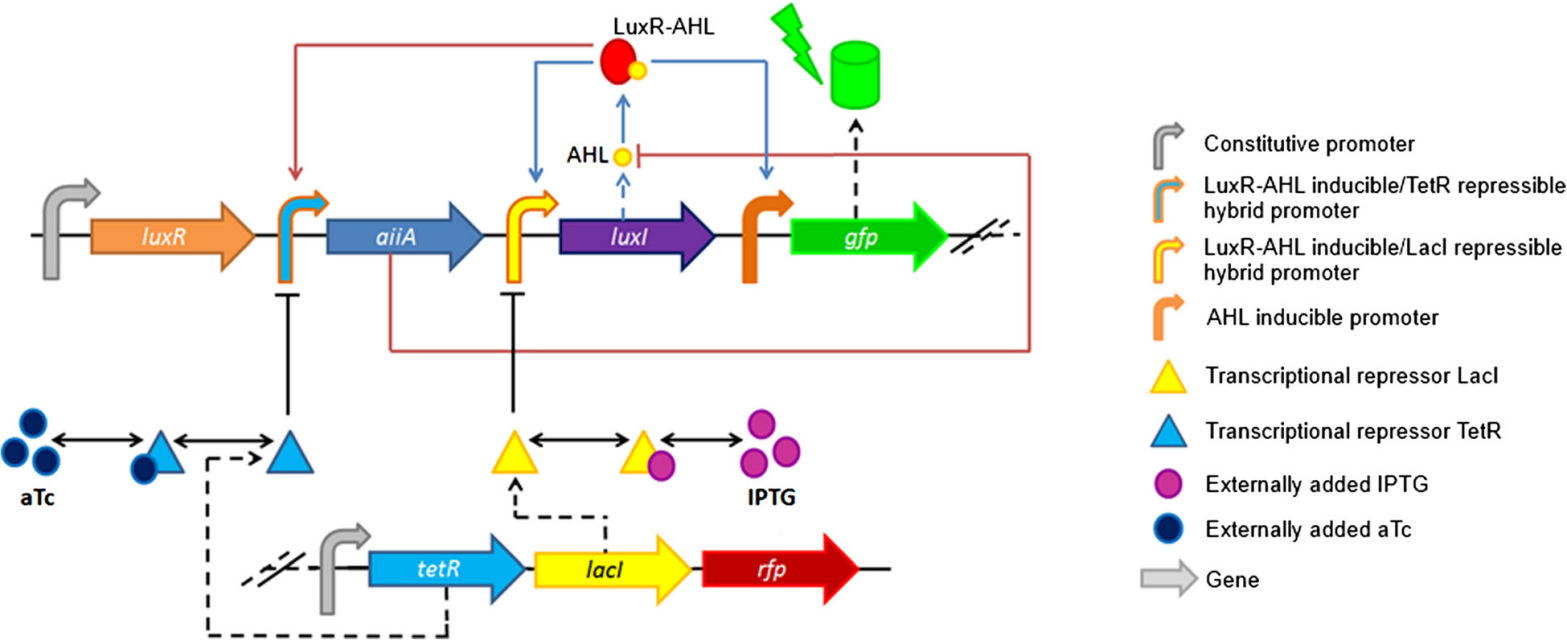
**Circuit expanded with repressors enables tuning of the feedback kinetics.** The transcriptional repressors TetR and LacI and the red fluorescent reporter mCherry are expressed as a contiguous transcript under control of the araC/pBAD promoter. TetR and LacI repress the transcription of aiiA and luxI, respectively. The presence of the inducer molecules aTc and IPTG relieve the repression as a function of their intracellular concentrations, effectively modulating the strength of the oscillating components’ transcriptional feedback.

In the basic circuit described before, cell density is one of only two system parameters which can be manipulated, the other being the AHL-removal rate (i.e. medium replacement). All other parameters, including the maximum transcription rates of *luxI* and *aiiA,* are inaccessible barring the introduction of further regulatory elements. This constraint was circumvented by implementing “tuners”, that is, constitutively expressed LacI and TetR transcription factors, which respectively repress the transcription of *luxI* and *aiiA.* The extent of the repression is dependent on the intracellular concentration of the corresponding inducer molecules isopropyl β-D-1-thiogalactopyranoside (IPTG) for LacI, and anhydrotetracycline (aTc) for TetR. The expanded circuit including the tuners is schematically represented in Figure 2.

The circuit represented in Figure 2 shows the oscillatory circuit with the aforementioned modifications: (i) elimination of two redundant copies of the sequences encoding the transcriptional regulator LuxR, (ii) replacement of natural bidirectional quorum sensing promoters with synthetic hybrid promoters containing repressor binding sites, the activity of which can be independently controlled via chemical inducers and (iii) consolidation of all the circuit’s components, including the tuner module, into a single device conforming to the BioBrick assembly standard.

### Basic oscillator tested in a flow device

**Figure 3:**
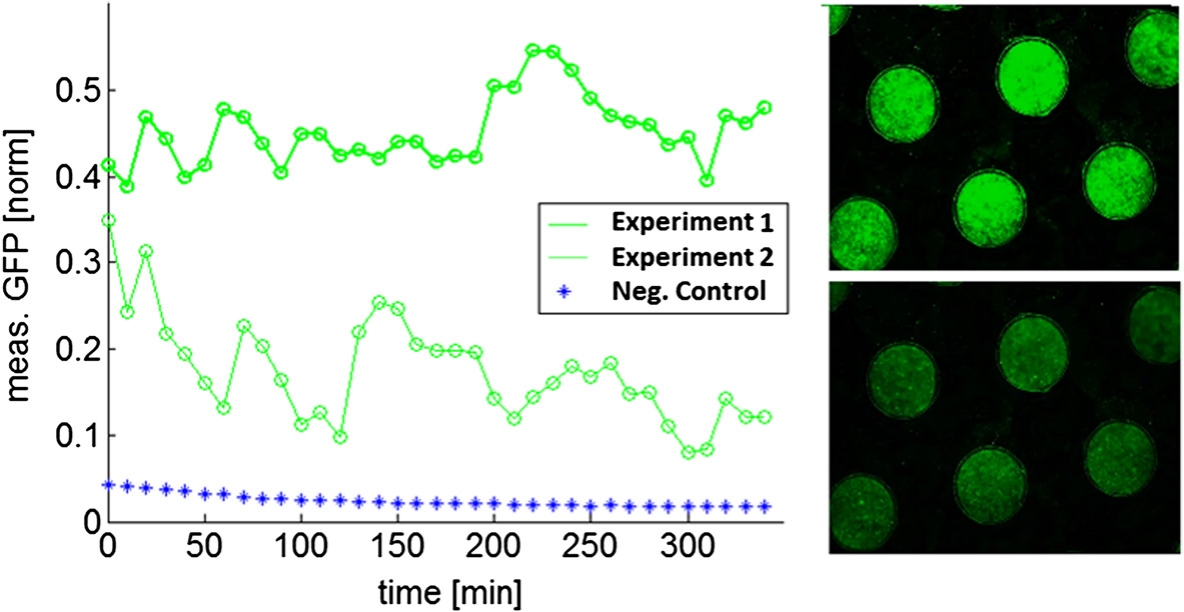
**Oscillatory GFP expression measured in flow device.** Left, measured oscillations in GFP expression of cells containing oscillator without the repressors and cells containing construct for constitutively expressed GFP as negative control. The plotted data represents the average intensity of a single well in the focus area per experiment. Right, cells grown in the wells of the microdish at high (top) and low (bottom) GFP expression levels.

To validate the functionality of the basic synchronized oscillator construct depicted in Figure 1, fluorescence microscopy measurements were taken of *E. coli* cells harbouring the plasmid grown in a microdish [19,20]. Time spans of 5 hours over which oscillations in GFP intensity were observed are depicted in Figure 3, showing the functionality of the oscillatory expression dynamics as compared to *E. coli* cells containing a construct with constitutively expressed GFP, as previously reported [19]. While synchronized oscillatory GFP expression was repeatedly observed, there were substantial differences in the measured frequencies and amplitudes between experiments. The measurements furthermore appear to be noisier than previously published results in which a similar genetic circuit was tested using a microfluidic chip. The specific geometry of this platform may lead to less consistent gene expression than observed in set-ups with more defined fluid control and smaller cell retention spaces [14]. Since our measurements were performed under zero-flow conditions (no media replacement), non-enzymatic AHL-removal only occurred via diffusion, resulting in a net accumulation of AHL. This eventually led to a steady state in which the enzymatic degradation was not sufficient to reset the system. It is reasonable to assume that once a critical AHL threshold is reached, further fluctuations in AHL concentration no longer significantly affect transcriptional dynamics due to saturation of LuxR. This may explain both the relative shallowness and shorter duration of the oscillations compared to a setup in which AHL was actively removed via controlled medium replacement [14]. However, another result of the accumulation of AHL was that, surprisingly, synchronization was not limited to populations within individual wells, but was observed among all of the wells within the measured area (approx. 0.63 × 0.9 mm). Since AHL is a small molecule, it is likely that it can diffuse through the porous aluminium oxide matrix [19]. This allows wells of different cell densities to be synchronized, as the AHL concentration in the medium below mimics a high cell density even in wells of lower cell densities. However, it is unclear whether the AHL concentration in the medium below the matrix is also homogenous.

Work published by Prindle et al. [21] demonstrated synchronization of cells trapped in microfluidic chambers across a distance of 5 mm via diffusion of H_2_O_2_. Our findings are consistent with this to the extent that synchronization was observed across various spatially separated sub-populations. However, an important difference is that in our system populations are solely coupled by AHL diffusion, and therefore dependent on a fluid medium to travel. The diffusion kinetics of different signaling molecules therefore need to be taken into account when designing chemically coupled regulatory systems.

### Computational simulations illustrating differential regulatory dynamics

**Figure 4:**
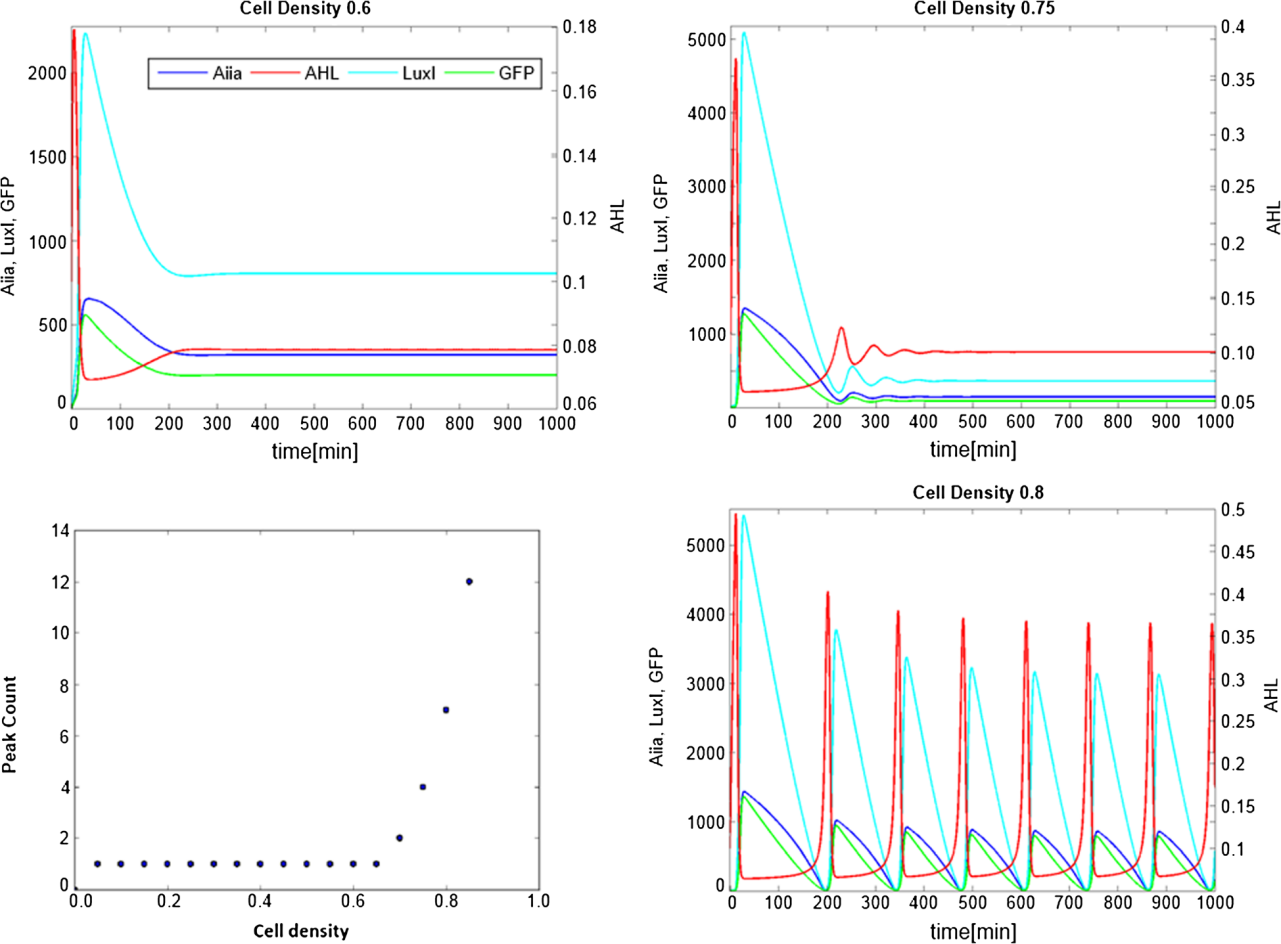
**Correlation between the cell density and the onset of synchronized oscillatory behavior across a population of cells.** As LuxI (cyan) is produced more rapidly than AiiA (dark blue), AHL (red) will initially peak to a high level before being degraded and reaching a low basal level. At low cell densities, the AHL level is not enough to induce synchronized oscillatory behavior across a population of cells. At higher cell densities however there will be enough AHL in the system to coordinate the dynamic expression of the oscillatory construct within the cells, thus sustained oscillations in GFP levels will be observed.

Deterministic simulations based on a dynamic model consisting of five delay differential equations were performed to illustrate the differences between the tunable and non-tunable circuits. In absence of the repressors (LacI and TetR both set to 0), the inducer molecules IPTG and aTc have no effect on gene expression. In this case the system’s dynamics are governed solely by the quorum sensing machinery, which operates as a function of cell density via the inducer molecule AHL, which diffuses freely throughout the system and thus synchronizes the population. At cell densities below 0.65, where 0.88 is the theoretical maximum constraint given by the model, there is not enough AHL present to induce synchronization and thus no sustained oscillatory GFP expression is observed. The result is a single high peak before settling into a steady state. After passing a cell density of 0.65, the cells are able to produce enough AHL to cause damped oscillations in GFP expression. At cell densities greater than 0.75, the AHL threshold value is reached and sustained oscillations across the population of cells can emerge. The oscillations increase in frequency as they approach the maximum cell density. In all cases, the waveform is that of a relaxation oscillator, which is consistent with the previously described experimental results. The relationship between the onset of oscillations and the cell density is depicted in Figure 4.

**Figure 5:**
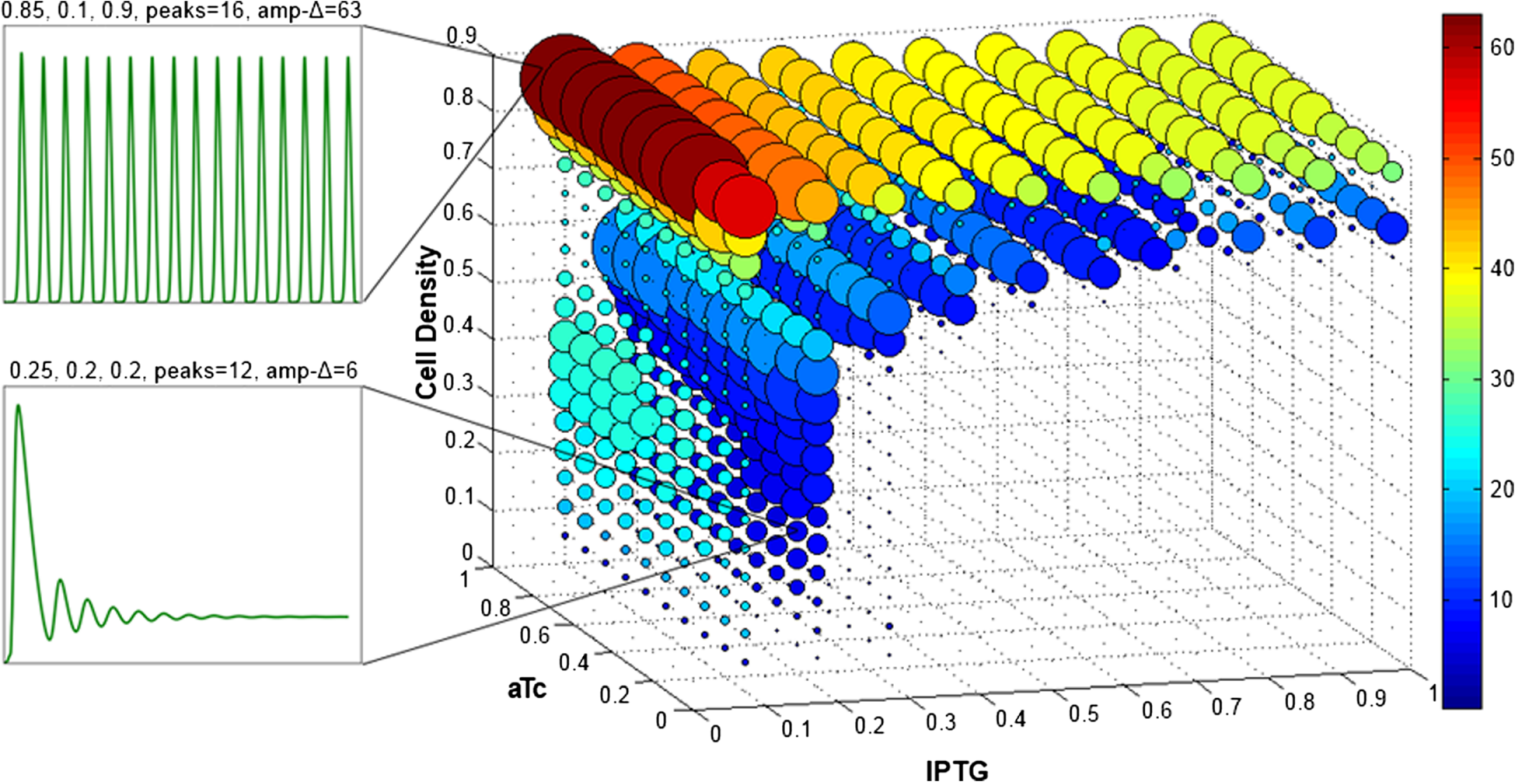
**5-dimensional scatterplot representing the result of 2178 simulations.** Inducer molecule concentrations and cell density are the input variables. The radius of each circular marker is proportional to 2^n^, where n is the number of peaks in a given simulation and can be interpreted as a measure of frequency. This nonlinear scaling emphasizes the differences between high-frequency oscillations, and prevents them from being occluded by the far more numerous low-frequency oscillations. The color of each marker represents the sum of the differences between all maxima and minima normalized against the average of all values in a given simulation. Thus the color serves as a measure of amplitude.

When the repressors LacI and TetR are present, the system’s expression dynamics are influenced by the concentrations of the inducer molecules IPTG and aTc. The results of 2178 simulations over 1000 time points are represented as a 5-dimensional scatterplot in Figure 5. The Cartesian x,y and z coordinates represent IPTG, aTc, and cell density, respectively. The size and color of the circular markers are each determined by scalars representing metrics that capture the essential properties of the waveform generated by the simulated GFP expression. An increase in marker size corresponds to an increase in frequency. The color change from blue to red corresponds to an increase in the normalized amplitude metric.

It is clear from this representation that the relationships between the system inputs and the resulting waveforms are non-linear, giving rise to a number of localized trends within the 5-dimensional space. The most obvious feature is the relative sparseness of the space for IPTG values greater than 0.3 and cell density values smaller than 0.6. This space is populated exclusively by damped oscillations with few peaks. It is noteworthy that for small values of IPTG the frequency steadily increases as a function of cell density before decreasing (around 0.6 for IPTG = 0.2) and subsequently increasing again.

Furthermore, cell density is strongly correlated to the amplitude metric. This is related to the fact that sustained oscillations score far higher in this metric than damped oscillations, and sustained oscillations only occur outside the sparse parabolic region that spans the majority of the volume.

**Figure 6:**
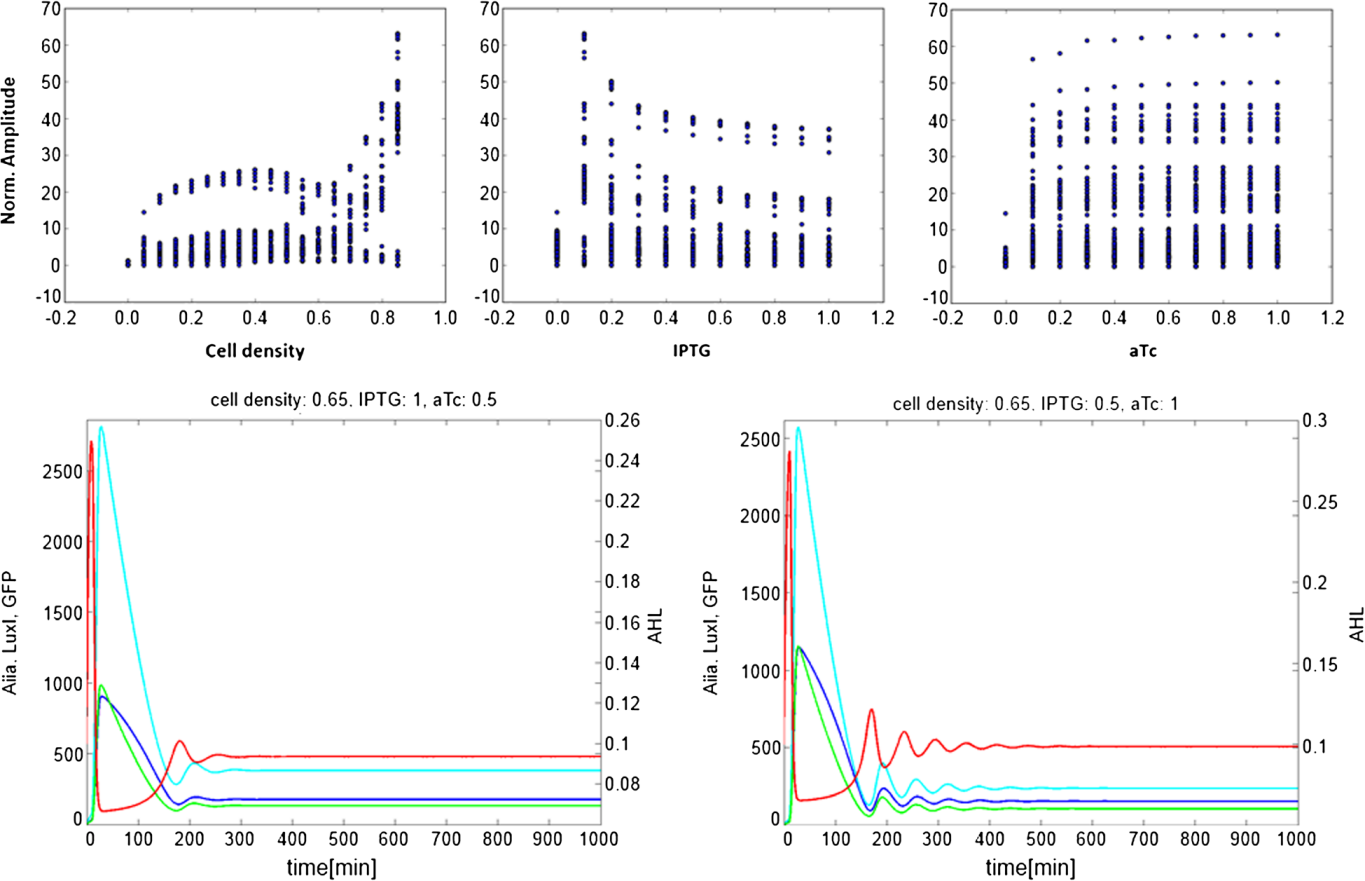
**Top: 2-dimensional scatterplots separately comparing the normalized mean amplitudes to the 3 input variables.** The amplitude metric varies the most as a function of cell density, and the least as a function of aTc. Bottom: Simulations comparing the effects of different ratios of IPTG and aTc.

The relationship between the amplitude metric and the input variables is best seen as a 2-dimensional scatterplot (Figure 6, top). Values below 20 (dark blue in the 5-dimensional plot) correspond to damped oscillations in all cases. The distribution is inhomogeneous when the amplitude is plotted against the cell density. It is more homogeneous when plotted against the IPTG level, and even more so when plotted against the aTc level. The more homogeneous the plot, the less effect changes in the variable have on the amplitude. It might seem counter-intuitive that increasing levels of IPTG do not correspond to an increasing range of cell densities in which oscillations occur, as increased IPTG results in decreased repression of AHL production. We propose that the predicted dynamics are due to the difference in the time it takes for the enzymes to become active [14]. Since the maturation of the AHL-degradase takes longer than that of the AHL-synthase, AHL can accumulate and reach a saturation threshold which will effectively produce a steady state. This can be seen in the comparison between mirrored ratios of IPTG and aTc (Figure 6, bottom). When IPTG exceeds aTc, the system requires a higher cell density to oscillate than in the reverse case.

**Figure 7:**
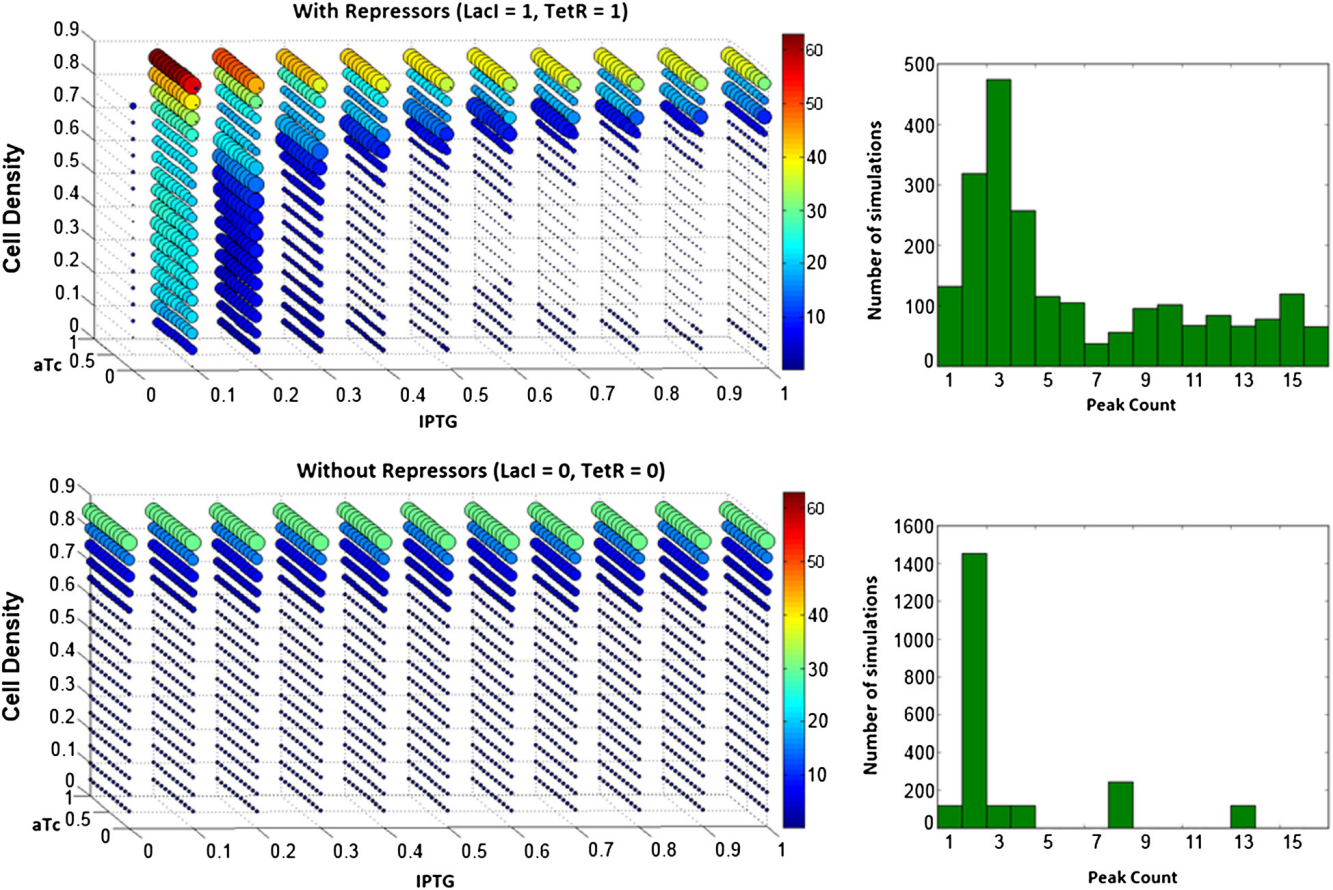
**Comparison between the simulated circuit with and without repressors.** Simulated dynamics of the tunable and non-tunable circuits demonstrates that the introduction of tuners greatly enhances the range of cell densities under which sustained oscillations can occur. Maximum achievable amplitude is also substantially higher, as is the range of frequencies that can be generated.

A comparison between the tunable and non-tunable system is shown in Figure 7. The 5-dimensional scatterplots show that the non-tunable system can only oscillate at cell densities above 0.6, whereas the tunable system oscillates at all simulated cell densities when IPTG is below 0.3. The maximum amplitude is around 60 for the tunable, and around 30 for the non-tunable circuit. Frequency variability is visualized by histograms, which show that the tunable system can produce a waveform with any number of peaks between 0 and 16, whereas the non-tunable system is comparatively limited. These simulations indicate that while damped oscillations (dark blue) still make up the vast majority of waveforms produced by the tunable circuit, this system is capable of a significantly more diverse behavior than the original non-tunable design. It is clear that the changes made to the original circuit have a substantial effect on the resulting protein expression dynamics. The introduction of mathematical expressions representing chemical inducer molecules and corresponding orthogonal transcription factors revealed an unexpected range of non-obvious relationships between the system components.

## Conclusion

The aim of this study was to test the functionality of a refactored synchronized transcriptional oscillator and to investigate whether its reliability and utility could be enhanced by the introduction of chemically inducible repressors. The functionality of the basic circuit, assembled from BioBrick parts, was verified experimentally using a custom experimental platform. These experiments revealed synchronization at an unexpected scale between spatially separated but chemically linked populations of bacteria. Computational simulations of the tunable circuit design revealed a rich landscape of non-linear relationships between the oscillatory behavior of the circuit and the control variables. The simulations suggested that, while cell density is the primary determinant of gene expression dynamics in this system, the ability to tune transcriptional feedback kinetics via inducer molecules substantially broadens the range of waveforms that this circuit can generate. Assuming that the model upon which the simulations were based capture the actual dynamics, the tunable oscillator design described here should be highly versatile. While fluorescent plate reader experiments aimed at characterizing this circuit’s tunability repeatedly demonstrated dynamic gene expression, a lack of consistency between replicates was confounded by a low signal to noise ratio, ultimately yielding inconclusive results (data not shown).

These results offer a cursory glance at the type of methods that could be employed to study nonlinear transcriptional regulatory dynamics using this circuit. Future work on this system should aim to validate the model before exploring more rigorous analytical methods.

Due to its efficient single-plasmid design it also lends itself to the investigation of expression dynamics as a function of varying copy numbers using different plasmid backbones, or the effect of genomic integration. Such approaches could very well yield reliable, quantitative data if combined with advanced experimental platforms, such as fluorescence microscopy combined with microfluidics, fluorescence-based cell-sorting methods, or milliliter-scale continuous stirred-tank bioreactors. It is our hope that in the future, this circuit may be used by others as a tool for developing, and possibly benchmarking increasingly refined modeling approaches that shed light on the intricate and elusive properties of complex genetic circuits.

## Methods

### Circuit assembly

Both the basic and tunable oscillator circuits were assembled from BioBrick parts obtained from the Registry of Standard Biological Parts (http://www.partsregistry.org), listed in Table 1. Parts were assembled hierarchically, two at a time using the BioBrick standard assembly method [18,22]. The receiving plasmid with a pSB1A2 backbone was cut with either EcoRI and XbaI or with SpeI and PstI restriction enzymes. The insert was liberated from the donor plasmid by digestion with either EcoRI and SpeI or XbaI and PstI restriction enzymes. After digestion the fragments were separated via gel electrophoresis and subsequently isolated with a Qiagen Gel Extraction kit. The purified fragments were then ligated using T4 Ligase and used to transform chemically competent *E. coli* TOP10 cells using a heat shock protocol. After a recovery in SOC medium the cells were plated on LB agar containing either chloramphenicol or ampicillin and grown overnight at 37°C. Colony PCR was used to screen for successful transformants which were then used for the inoculation of 10 mL liquid cultures of LB and grown overnight at 37°C. The resulting composite BioBrick part was then isolated from these liquid cultures using a Qiagen miniprep kit.

**Table 1:** BioBrick parts

**Component**		**Function**	**Transcriptional control**		**Source**
LuxR	C0062	AHL-dependent TF	Constitutive	J23101	*Vibrio fischeri*
LuxI-LVA	C0061	AHL-synthase	Induced by AHL-LuxR Repressed by LacI	I751502	*Vibrio fischeri*
AiiA-LVA	C0060	AHL-degradase	Induced by AHL-LuxR, Repressed by TetR	K176000	*Bacillus thuringiensis*
GFP-LVA	J04031	Reporter molecule	Induced by AHL-LuxR	R0062	*Aequeora victoria*
TetR	C0040	Repressor of *aiiA*	araC/pBAD	I0500	*Escherichia coli*
LacI	C0012	Repressor of *luxI*	araC/pBAD	I0500	*Escherichia coli*
mCherry	J06504	Constitutive reporter molecule	araC/pBAD	I0500	*Discosoma sp.*
Backbone	pSB1A2	Cloning & expression vector	N/A	N/A	*Escherichia coli*

All components that were used to make the genetic circuits are listed with a generic name, BioBrick ID, and function, as well as their transcriptional regulation, promoter BioBrick ID, and the biological source of the part.

### Fluorescence measurements using microdish

Liquid cultures were made from single colonies which had grown on LB agar plates with ampicillin (50 μg/mL). The single colonies were grown over night at 37°C in 10-15 ml of LB ampicillin medium. The cultures were spun down and resuspended in 0.9% phosphate buffered saline (PBS), before inoculation in a custom made flow device [19] equipped with a microdish made from porous aluminium oxide containing 40 μm deep wells with a diameter of 180 μm [20]. Since LB-amp medium was supplied from below the microdish, the growth of bacteria was restricted to the wells there nutrients could be obtained via diffusion through the porous material at the base. The visual output was measured using an Olympus fluorescence microscope BX41 with an exposure time of 200 ms and 100 × magnification. Measurements were taken in a time interval of 10 minutes by a Mindstorms Lego robot (http://mindstorms.lego.com). Data analysis and processing were done with ImageJ 1.45 (http://rsbweb.nih.gov/ij/index.html) and MATLAB (http://www.mathworks.com).

### DDE model

The genetic circuit described above can be represented as a system of delay differential equations, which was adapted from Danino et al. [14] and expanded with Hill functions to represent the effect of the tuner repressors and their inducer molecules on the maximum expression level of the dynamically expressed components. The final model is presented as Equations 1, 2, 3, 4, 5.

The terms proportional to C_A_ (1) and C_L_ (2) represent the dependency of AiiA and LuxI expression on the cell density d. The hybrid promoters regulating *luxI* and *aiiA* were assumed to have the same response kinetics to LuxR-AHL as the natural lux promoter in the absence of the repressor proteins. To take possible differences between the hybrid promoters into account, the leakage constants for *aiiA* and *luxI* expression were replaced by the new leakage constants δ_1_ and δ_2_. The terms containing these leakage constants are the history functions present in the original model which consolidate the time delay resulting from gene transcription and translation into a single parameter τ. The term after that in equations (1) and (2) is the actual tuner term and represents the influence of the repressors on the system, which in turn is dependent on the presence of either IPTG for LacI or aTc for tetR, respectively. Equations (3) and (4) contain an additional term proportional to D, which shows the diffusion of AHL throughout the cells. Finally, the terms proportional to γ show the degradation of AiiA, LuxI and GFP in equations (1), (2) and (5), respectively.

In accordance with observations of most naturally occurring regulatory elements, all promoters are assumed to be “leaky”, and exhibit a basal expression level in the absence of activating TFs [13]. The model is applicable to both the basic circuit and the tunable one, as the model for the latter can be reduced to represent the former simply by setting the concentration of the repressors to 0.

Deterministic simulations were performed using the MATLAB dde23 solver in order to elucidate the relationship between inducer molecule concentrations and their effect on GFP expression relative to cell density. The input values were chosen to cover the entirety of the controllable input space, ranging from full repression (IPTG and aTc set to 0) to full induction (both IPTG and aTc set to 1) in steps of 0.1. The cell density was also iterated from 0 to 0.85 in steps of 0.05, resulting in a total of 2178 simulated conditions.

Technically the expression of TetR and LacI is not constitutive due to regulation by the AraC/pBAD promoter and its corresponding inducer molecule arabinose. However, it is treated as such for the purposes of this study.

## Appendix

(1) $$\begin{array}{l} \frac{\mathrm{dA}}{\mathrm{dt}}=C_{A}\cdot \left[1-{\left(\frac{d}{d_{0}}\right)}^{4}\right]\cdot \frac{\delta_{1}+a\cdot Hi_{t-\tau }^{2}}{1+k_{1}\cdot Hi_{t-\tau }^{2}}\cdot \frac{1}{1+\frac{\beta_{1}\cdot R_{A}}{1+\beta_{2}\cdot I_{A}}}\\ \;-\mathrm{\gamma A}\cdot \frac{A}{1+f\left(A+L+G\right)} \end{array}$$

(2) $$\begin{array}{l} \frac{\mathrm{dL}}{\mathrm{dt}}=C_{L}\cdot \left[1-{\left(\frac{d}{d_{0}}\right)}^{4}\right]\cdot \frac{\delta_{2}+a\cdot Hi_{t-\tau }^{2}}{1+k_{1}\cdot Hi_{t-\tau }^{2}}\cdot \frac{1}{1+\frac{\beta_{3}\cdot R_{L}}{1+\beta_{4}\cdot I_{L}}}\\ \;-\mathrm{\gamma L}\cdot \frac{L}{1+f\left(A+L+G\right)} \end{array}$$

(3) $$\frac{\mathrm{dHi}}{\mathrm{dt}}=\frac{b\cdot L}{1+k\cdot L}-\mathrm{\gamma H}\cdot \frac{A\cdot \mathrm{Hi}}{1+g\cdot A}+D\cdot \left(\mathrm{He}-\mathrm{Hi}\right)$$

(4) $$\frac{\mathrm{dHe}}{\mathrm{dt}}=-\frac{d}{1-d}\cdot D\cdot \left(\mathrm{He}-\mathrm{Hi}\right)$$

(5) $$\frac{\mathrm{dG}}{\mathrm{dt}}=C_{G}\cdot \left[1-{\left(\frac{d}{d_{0}}\right)}^{4}\right]\cdot \frac{\delta +\alpha \cdot Hi_{t-\tau }^{2}}{1+k_{1}\cdot Hi_{t-\tau }^{2}}-\gamma_{G}\cdot \frac{G}{1+f\left(A+L+G\right)}$$

Equations1, 2, 3, 4, 5. Set of delay differential equations representing the interactions between the circuit’s components. The terms representing time and cell-density dependent changes in AiiA (1), LuxI (2), and GFP (5) all have the same basic features. The main difference is that the maximum expression levels of AiiA and LuxI are limited by Hill functions that take the concentrations of their respective repressors and corresponding inducer molecules into account. In contrast, the expression of GFP is only dependent on the cell density and intracellular AHL concentration. Changes in intracellular AHL (3) are a function of LuxI and AiiA levels as well as a diffusion term. A = AiiA, L = LuxI, H_i_ = internal AHL, H_e_ = external AHL, C = production constant, d = cell density, δ = promoter leakage, γ = degradation constant, D = diffusion rate, τ = time delay.

## Abbreviations

GFP: Green fluorescent protein; aTc: Anhydrotetracycline; IPTG: Isopropyl-β-D-thiogalactopyranoside; FACS: Fluorescence-activated cell sorting; AHL: N-(3-oxohexanoyl)-homoserine lactone.

## Competing interests

The authors declare that they do not have any competing interests.

## Authors’ contributions

BMR and MCH designed the constructs. BMR, MCH, RvH and DIO assembled the constructs. BMR, DIO, MvP and FH designed the experiments. BMR, DIO, RvH and YvN performed the experiments and analysed data. DIO and RvH developed the model and performed the simulations. BMR and DIO analysed and visualized the simulation data. FH, MvP and VMDS supervised the project. BMR and DIO wrote the paper. All authors read and approved the final manuscript.
